# Malnutrition and associated factors among heart failure patients on follow up at Jimma university specialized hospital, Ethiopia

**DOI:** 10.1186/s12872-015-0111-4

**Published:** 2015-10-15

**Authors:** Hiwot Amare, Leja Hamza, Henok Asefa

**Affiliations:** Department of Internal Medicine, Jimma University, Jimma, Ethiopia; Department of Epidemiology and Biostatistics, Jimma University, Jimma, Ethiopia

**Keywords:** Malnutrition, Angiotensin converting enzyme inhibitors, Serum hemoglobin, Serum albumin, Heart failure in Ethiopia

## Abstract

**Background:**

Malnutrition and cachexia are serious consequences of numerous chronic diseases. Severe heart failure patients could be related with marked weight loss. Malnutrition is associated with poor prognosis among heart failure patients. Despite its implication, factors associated with malnutrition among heart failure patients in Africa and Ethiopia was not addressed. Hence, in this study we tried to determine factors associated with malnutrition among heart failure patients on follow up at Jimma University specialized hospital, Ethiopia.

**Methods:**

A cross-sectional study was done on 284 randomly selected heart failure patients. The nutritional status of the patients was assessed based on their serum albumin level (normal value 4–5 mg/dl) and triceps skin fold thickness. The data was analyzed using SPSS version 20.0. Multivariable logistic regression was used to identify factors associated with malnutrition among heart failure patients using SPSS 20.0.

**Results:**

Based on serum albumin and triceps skin fold thickness, 77.8 % of patients were malnourished. Mean age of the patients was 48.3 ± 15.9 years. The commonest cause of heart failure was ischemic heart disease (34.9 %). Hypertension (36 %) was the commonest co morbid disease. Forty four percent of patients had New York heart association functional class II heart failure. Serum hemoglobin (AOR = 0.77, 95 % CI: 0.67–0.92) was found to be significantly associated with nutritional status of heart failure patients. As serum hemoglobin increases by 1gm/dl, the risk of malnutrition decreased by 15 % (*P* value = 0.03).

**Conclusions:**

The majority of patients were malnourished. A higher hemoglobin concentration was associated with reduced odds of being malnourished.

## Background

Globally, cardiovascular diseases are the leading cause of death. In 2012, it had caused an estimated 17.5 million deaths, which accounted 31 % of all global deaths. With some variation in epidemiology due to the factors associated with life style, behavioral risk factors, genetic and racial differences; there is a rise in the burden of cardiovascular diseases in low and middle income countries. Over three quarters of cardiovascular deaths take place in low- and middle-income countries such as Asia and Sub-Saharan Africa [1].

A systematic review performed on heart failure and diabetes in Sub-Saharan Africa, showed that heart failure accounts for over 30 % of hospital admission in specialized cardiovascular units and 3–7 % in general internal medicine [2]. There are no published data showing the national prevalence of heart failure from Africa and Ethiopia.

Cachexia and malnutrition are serious complication of numerous malignant and non malignant diseases like chronic obstructive lung disease chronic kidney disease and severe heart failure. Malnutrition in heart failure is associated with loss of muscles, fat and bone mass. Its causes can be due to decreased intake, increased loss of nutrients, increased metabolic rate and cytokine dysfunction involving tumor necrosis factor-alpha (TNF-α), cortisol, epinephrine, renin as well as aldosterone. Drugs used in treatment of heart failure such as angiotensin converting enzyme inhibitors can prevent malnutrition and sarcopenia in heart failure [3–12].

The assessment of malnutrition in heart failure might be done by anthropometric and biochemical tests. Numerous studies had used BMI, mid upper arm circumference (MUAC), calf circumference and triceps skin fold thickness [13]. A study from Spain that assessed the usefulness of body mass index to characterize the nutritional status in patients with heart failure concluded that BMI does not indicate true nutritional status in HF. Among nutritional indicators, triceps skin fold is a well standardized method of assessment of malnutrition for age and sex in heart failure [14].

Serum albumin is one of the biochemical tests to assess nutritional status in heart failure used in numerous studies. Serum albumin is used in assessment of protein malnutrition without calorie malnutrition in which serum albumin becomes low without affection of anthropometric measurements [13]. Using serum albumin has its own limitations such as its variation with several non nutritional factors such as status of hydration (states of over hydration lead to overestimation and dehydration leads to underestimation of serum albumin). Albumin is also a negative acute phase reactant therefore; states of inflammation, infection and malignancies can also decrease its serum level [15, 16].

Malnutrition was found to be associated with worsening of symptoms and poor prognosis. Multiple European studies showed malnourished heart failure patients are weaker and fatigue earlier [11, 17] and a BMI of 27–29 was found to be ideal in patients with heart failure, with mortality increasing either side of this range [18, 19].

Heart failure with hypoalbuminemia, as indicator of malnutrition, was found to be associated with higher New York heart association (NYHA) functional class, elevated serum blood urea nitrogen and C-reactive protein. It is also linked with lower serum hemoglobin, sodium and cholesterol [20, 21]. Vitamin and mineral deficiencies could further increase mortality in these malnourished patients [22]. Studies done by European and American researchers also showed that malnutrition and body wasting were also associated with wasting of the left ventricle [23].

There are hardly any studies done on malnutrition in heart failure in Africa and to our knowledge there are no studies which assessed both prevalence of malnutrition and associated factors among heart failure patients in Africa and Ethiopia. Hence, this study was conducted to assess determinant factors affecting malnutrition among heart failure patients.

## Methods and patients

### Settings

The study was done on heart failure patients that were on follow up at Jimma University specialized hospital (JUSH) located in Jimma town south west Ethiopia. The hospital serves as a teaching and referral hospital with a catchment of 15 million people. Heart failure patients are followed at a weekly cardiac clinic.

### Design and data collection

A hospital based cross sectional study was conducted from November 2013 to June 2014. The list of patients who were on follow up during the study period was obtained from the cardiac clinic and using a simple random sampling technique a sample of 310 patients who fulfill the inclusion criterion were selected to be included in the study. Edematous patients and those who didn’t consent for the study were excluded because edematous state can lead to over estimation of serum albumin [15, 16]. The required sample size was calculated using a single population proportion formula, assuming the prevalence of malnutrition among heart failure patients to be 50 % to obtain maximum sample size since this prevalence is unknown. After applying finite population correction the required sample size to conduct this study was 310 patients. Ethical clearance was obtained from the Ethical Committee of Jimma University college public health and medical sciences (Ethics reference number – RPGC/266/2013). Verbal consent was also obtained from patients included in the study.

Data was collected by the primary investigator and trained clinical nurses. Socio demographic information was collected using a structured questionnaire from patients by interview. The patients charts were reviewed to obtain clinical information such as treatment course, New York functional class, length of follow up, comorbidities and cause of heart failure (from echocardiography result). Due to the lack of facilities for coronary angiography which is the golden standard for the diagnosis of ischemic heart disease, the diagnosis of ischemic heart disease was based on clinical parameters that included previous history of ischemia/infarction, abnormal biomarkers, ECG changes of old Myocardial infarction and echocardiographic findings of wall motion abnormalities, systolic dysfunction assessed by depressed ejection fraction of less than 50 %. Ejection fraction was calculated with the formulae systolic volume divided by end diastolic volume. The diagnosis of rheumatic valvular heart disease was based on echocardiography that is identified if it showed multi valvular lesions, valvular regurgitation or stenosis with either thickening or valve retraction. Hypertensive heart disease was diagnosed if there was left ventricular hypertrophy or depressed ejection fraction without wall motion abnormality on patients diagnosed with hypertension. Family support was defined as monetary support and support during home activities such as cooking. The New York functional class was assessed based on the following categorization. Class I –Patients with cardiac disease but without resulting limitation of physical activity. Ordinary physical activity does not cause undue fatigue, palpitations, dyspnea, or anginal pain. Class II –Patients with cardiac disease resulting in slight limitation of physical activity. They are comfortable at rest. Ordinary physical activity results in fatigue, palpitation, dyspnea, or anginal pain. Class III - Patients with cardiac disease resulting in marked limitation of physical activity. They are comfortable at rest. Less than ordinary activity causes fatigue, palpitation, dyspnea, or anginal pain. Class IV - Patients with cardiac disease resulting in inability to carry on any physical activity without discomfort. Symptoms of heart failure or the anginal syndrome may be present even at rest. If any physical activity is undertaken, discomfort is increased [24].

Anthropometric measurements such as weight and height and triceps skin fold thickness were measured. Weight was measured by a standing weight scale and height was measured with a height scale after daily calibration before starting data collection. Triceps skin fold thickness was measured by Lange skin fold caliper beta technology by staff trained in anthropometric/skin fold assessment. Triceps skin fold thickness is chosen as it is most used in chronic heart failure patients and is well standardized based on age and sex [14]. The mean of three measurements of each of the anthropometric assessments were taken and was reported as the final measurement.

Blood tests for serum albumin, hemoglobin, mean corpuscular volume; total lymphocyte count and platelet count were performed for biochemical assessments of nutrition for each patient on the same day of interview. A 5 ml blood sample was taken from the cubital fossa, 2.5 ml was put in an EDTA tube for complete blood count and 2.5 ml was put in a tube with no anticoagulants for serum albumin determination. Serum albumin was determined by HORIBA medical™ ABX Pentra 400 with use of appropriate human serum controls. Hemoglobin, mean corpuscular volume (normal value = 79-93 fl) [25] and total lymphocyte count (normal value = 710–4530 mm^2^) [25] and platelet count (165–415 × 10^3^/mm^3^) [25] were determined by complete blood count determination by Cysmex KX21 machine by the appropriate reagent after daily calibration. All results were expressed to the nearest 0.1 decimal place. Nutritional status was assessed based on serum albumin level and triceps skin fold thickness. Accordingly, a patient was considered as malnourished if serum albumin level was below 4 mg/dl and triceps skin fold thickness below the 50th percentile for age and sex [25, 26].

### Data analysis

Data were entered, edited, cleaned, coded and analyzed using SPSS version 20.0 for Windows. First, descriptive analysis was done. Then bivariate analysis was done to identify factors associated with nutritional status. Those variables in the bivariate analysis with P –value < 0.25 were considered as candidates to be included in the multivariable logistic regression model. The multivariate logistic regression was performed by the backward stepwise variable selection method with probability of removal 0.15. The adequacy of the model was checked by using Hosmer and Lemeshow goodness-of-fit test.

## Results

A total of 284 patients had participated in this study. Ten patients refused to participate in the study and 16 patients had incomplete charts, which made the response rate to be 91.6 %.

### Socio-demographic characteristics of patients

Among the patients, 162 (57.0 %) of them were females. Mean age of the patients was 48.3 ± 15.9 years. One hundred ninety two patients (67.0 %) were married. One hundred seventy one patients (60.2 %) were illiterate. One hundred twenty five patients (44.0 %) were unemployed. More than 49 % have no income and are dependent on their family and for those with income the median monthly income of the patients was 100 birr (5 U.S. Dollars) (Table 1).

**Table 1:** Socio-demographic characteristics of heart failure patients, JUSH, Ethiopia, from November 2013 to June 2014

Patient characteristics	Number	(%)
		Total (*N* = 284)
Sex		
Male	122	43.0
Female	162	57.0
Educational status		
Illiterate	171	60.2
Literate	113	39.8
Marital status		
Single	31	10.9
Married	192	67.6
Divorced	25	8.8
Widowed	36	12.7
Occupation		
Employed	159	56.0
Unemployed	125	44.0
Family support		
Patients with family support	272	95.8
Patients without family support	12	4.2

### Clinical profile

The commonest cause of heart failure was ischemic heart disease (34.9 %). The median length of follow up for heart failure was 36 months. From the 144 patients (50.7 %) that had comorbidity with heart failure, the commonest comorbidity was hypertension seen in 102 patients (70.8 %). One hundred ninety three patients (64.3 %) had no admission in the past 12 months. According to the NYHA assessment for functional class for heart failure patients, 123 (43.5 %) patients were categorized to NYHA functional status of Class II.

Ninety one percent of patients were taking more than one drug at a time; 179 patients (63 %) were on ACE-inhibitors; 50 patients (17.6 %) were on Spironolactone; 150 patients (51.1 %) were taking beta blockers (Table 2).

**Table 2:** Clinical profile of heart failure patients, JUSH, Ethiopia, from November 2013 to June 2014

Clinical profile	Frequency	%
		Total (*N* = 284)
Cause of heart failure		
Ischemic heart disease	99	34.9
Rheumatic valvular heart diseases	83	29.2
Hypertensive heart failure	69	24.3
Cardiomyopathy	25	8.8
Others	8	2.8
New York heart association functional class		
Class I	10	3.5
Class II	123	43.5
Class III	99	35.0
Class IV	51	18.0
Comorbid illness		
No Comorbid illness	140	49.5
Hypertension	102	36
Chronic kidney disease	17	6
Lung diseases	12	4.2
Others	12	4.2
Admission in the past 12 months		
Yes	106	37.3
No	178	62.7

### Nutritional assessment

The current mean weight of the heart failure patients included was 53.9 ± 11.9 kg. Thirty six patients (12.7 %) reported that they have noticed weight loss in the past 6 months. Twenty three patients had reported the amount of weight loss in the interview and six patients couldn’t specify the amount of weight loss. The rest did not report any weight loss in the past 6 months. For those patients who had reported objective weight loss, the mean weight lost was 4.5 ± 3.7 kg. The current median body mass index was 19.6 ± 3.7 kg/m^2^.

With laboratory investigations conducted from blood samples of patients, 154 patients (54.6 %) had normocytic, 96 patients (34.0 %) had macrocytic mean corpuscular volumes and 32 patients (11.3 %) had microcytic mean corpuscular volumes. The mean serum hemoglobin level was 12.6 ± 2 gm/dl. With total lymphocytic count, 97.5 % had normal values. Two hundred forty two patients (85.2 %) had normal platelet count.

Based on serum albumin and triceps skin fold thickness, 221 patients (77.8 %) were malnourished.

### Factors associated with malnutrition

Logistic regression model was used to identify factors associated with malnutrition among heart failure patients. Serum hemoglobin (AOR = 0.85, 95 % CI: 0.74–0.98) was found to be significantly associated with the nutritional status of heart failure patients at 5 % significance level. As serum hemoglobin increases by 1gm/dl, the risk of malnutrition decreased by 15 % (*P* value = 0.03) (Table 3).

**Table 3:** Variables associated with malnutrition among heart failure patients, JUSH, Ethiopia, from November 2013 to June 2014

Clinical profile	Adjusted OR	(95 % confidence interval)	*P*-value
Serum hemoglobin	0.85	0.74–0.98	0.02

-2 Log likelihood = 295.724

Cox & Snell R Square = 0.017

Nagelkerke R Square = 0.026

Hosmer and Lemeshow Test, *x* ^2^ = 9.5, P = 0.3

The variables included in the multivariable logistic regression were diuretic use, ACE –I use, cause of heart failure, frequency of admission in the past 1 year, New York heart association functional class, serum hemoglobin level, total lymphocyte count

## Discussion

This study revealed younger heart failure patients as compared to a multicenter European study (mean age = 67 years) [27] and a study from America (mean age = 61.2 years) [22] but in line with systematic reviews done by Kengne et al. and Sliwa et al. showed African patients were relatively younger as compared to their counterparts in the rest of the world (mean age = 52 years) [2, 28]. The THESUS-HF registry, which recruited African patients with acute decompensated heart failure, also showed a mean age of 52 years which is comparable to our study [29]. The early onset of heart failure in African population can be explained by the major contribution of rheumatic heart disease as a cause (second common cause of heart failure in this study) and the early onset and high prevalence hypertension in Africa as compared to European and American populations [2, 30, 31].

This study identified ischemic heart disease and rheumatic valvular heart disease to be the two common causes of heart failure respectively. This is comparable with studies from the developed countries but unexpected when compared to studies from developing countries and sub-Saharan Africa; that showed rheumatic valvular heart disease to be the most common etiology of heart failure [2] . This disparity could be due to a possible demographic transition [32], a rise in the prevalence of type 2 diabetes mellitus in Africa and Ethiopia [33] and high prevalence of hypertension in this study population. The unavailability of golden standard methods of diagnosis of ischemic heart disease in our set up can lead to over diagnosis.

Hypertension was the commonest co morbid disease (36 %) in this study, which is in line with a study done in American by Nicol et al., which showed prevalence of hypertension to be 44 % [34]. The lower prevalence of hypertension in our study could be explained by the younger patients seen in this study as compared to the study by Nicol et al. (62.8 years) as old age is associated with higher prevalence of hypertension [25]. Our result is also in line with other studies from sub-Saharan Africa, which reviewed data on heart failure and diabetes, identified hypertension to be the commonest comorbidity [2].

Based on serum albumin and triceps skin fold thickness, we have observed a high rate (77.8 %) of malnutrition. This finding is higher than a study done in United Kingdom by Anker et al. and United States by Mancini et al., which showed prevalence of cardiac cachexia to be 16 % (assessed by weight loss of >7.5 % in the past 6 months) and 24 % (based on serum albumin) among heart failure patients respectively [35, 36]. This disparity could be due to the difference in the method of assessment of malnutrition between the two studies or higher prevalence of malnutrition in the general Ethiopian population as compared to American population [37, 38]. The prolonged length of follow up could also make the prevalence of malnutrition to be higher due to progression of heart failure. The treatment of heart failure in this study population is suboptimal. Though not directly studied, we speculate that the suboptimal treatment of heart failure in our set up could be due to inability of patients to afford the drugs required due to low income (median monthly income is 5 USD); which could lead to rapid progression of heart failure and in turn explain the higher prevalence of malnutrition in our study [11] . Only taking a subset of heart failure patients with cardiomyopathy, another American study showed the prevalence of malnutrition to be about 50 % that may not be directly comparable with our study which included various causes of heart failure which contribute to higher prevalence of malnutrition [39].

In our study, the odds of malnutrition were higher in those who had lower serum hemoglobin level, this is in line with current knowledge [3, 24, 25]. It is well known that anemia has a strong impact on NYHA functional class in heart failure [40]. A Canadian study identified anemia by itself had a strong impact on clinical outcome of heart failure patients with higher number of anemic patients fitting to a higher functional class of heart failure [39]. Moreover, an Iraqi study done to assess hypoalbuminemia as a predictor of survival in systolic heart failure also showed that hypoalbuminemia was associated with a higher functional class of heart failure and lower serum hemoglobin level [21, 40].

With limitations of recall bias for subjective weight loss reporting and incomplete patient charts, the cross sectional study design which has a short coming of identifying determinant factors and causal relationships between multiple factors, the absence of a control arm, the unavailability of invasive investigations to confirm the cause of heart failure such as coronary angiography in the our setup; for the further assessment of other nutritional parameters such as body density measurement by using body plesthmography, assessment of vitamin and trace elements, assessment of other acute phase reactants like C reactive protein and pre albumin, assessment for concomitant other organ dysfunctions, and the inability to control for socio demographic parameter of the study participants which can affect nutritional status; serum hemoglobin was found to be associated with the nutritional status of heart failure patients.

## Conclusions

In conclusion, heart failure patients in our setup are relatively young. The major cause of heart failure was ischemic heart disease. Hypertension was the commonest co morbid disease associated with heart failure. Majority of heart failure patients were malnourished. Serum hemoglobin was found to be associated with nutritional status of heart failure patients.

We recommend that multicenter and case control studies with superior nutritional assessment methods should be done to further elaborate the association of serum hemoglobin with nutritional status of heart failure patients in Ethiopian population.

## Abbreviations

ACE –inhibitor: Angiotensin converting enzyme inhibitors
DALYs: Disability-adjusted life years
EDTA: Ethylenediaminetetraacetic acid
JUSH: Jimma University specialized hospital
TNF-α: Tumor necrosis factor alpha
USD: U. S dollars
